# Kinematic aspects of trunk motion and gender effect in normal adults

**DOI:** 10.1186/1743-0003-7-9

**Published:** 2010-02-15

**Authors:** Chin Youb Chung, Moon Seok Park, Sang Hyeong Lee, Se Jin Kong, Kyoung Min Lee

**Affiliations:** 1 Department of Orthopedic Surgery, Seoul National University Bundang Hospital, 300 Gumi-Dong, Bundang-Gu, Sungnam, Kyungki 463-707, Korea; 2 DooRee Motion Research Center, 223-17 Jamsilbon-Dong, Songpa-Gu, Seoul, 138-863, Korea

## Abstract

**Background:**

The purpose of this study was to analyze kinematic trunk motion data in normal adults and to investigate gender effect.

**Methods:**

Kinematic trunk motion data were obtained for 20 healthy subjects (11 men and 9 women; age from 21 to 40 years) during walking a 9 m long lane at a self selected speed, namely, motions in the sagittal (tilt), coronal (obliquity), and transverse (rotation) planes, which were all expressed as motions in global (relative to the ground) and those in pelvic reference frame (relative to pelvis), i.e., tilt (G), obliquity (G), rotation (G), tilt (P), obliquity (P), rotation (P).

**Results:**

Range of tilt (G), obliquity (G) and rotation (G) showed smaller motion than that of tilt (P), obliquity (P) and rotation (P), respectively. When genders were compared, female trunks showed a 5 degree more extended posture during gait than male trunks (p = 0.002), which appeared to be caused by different lumbar lordosis. Ranges of coronal and transverse plane motion appeared to be correlated. In gait cycle, the trunk motion appeared to counterbalance the lower extremity during swing phase in sagittal plane, and to reduce the angular velocity toward the contralateral side immediate before the contralateral heel strike in the coronal plane.

**Conclusions:**

Men and women showed different lumbar lordosis during normal gait, which might be partly responsible for the different prevalence of lumbar diseases between genders. However, this needs further investigation.

## Background

Trunk motion has not attracted much attention from those interested in three dimensional gait analysis, because this motion is relatively small and is generally thought to be passive and to depend on lower extremity motion. However, some recent studies have shown that trunk posture and motion can influence gait patterns of the lower extremity [1] and alter energy expenditure in the pathologic gait compared to a normal gait [2]. Moreover, the role of trunk motion in balance and proprioceptive function in gait [3,4] is being investigated by studying pathologic gait in patients with neurological, vestibular, or musculoskeletal diseases [5-7]. However, three dimensional gait analysis studies that have focusing on normal trunk motion have been somewhat limited, and as far as we know, no study has examined gender associated differences in trunk motion. We undertook this study to identify the kinematic aspects of normal trunk motion using three dimensional gait analysis and to determine whether the trunk motions of men and women are different, which may provide us with a possible explanatory clue for the different prevalences of spinal diseases between genders [8,9].

## Methods

### Inclusion criteria and data acquisition from three dimensional gait analysis

This study was approved by the institutional review board at our institute. Healthy adult volunteers, without musculoskeletal, neurological or cardiopulmonary disorders that could potentially have affected normal gait, were included in this study. Anthropometric parameters, such as, height, weight and BMI were recorded. Those volunteers who deviated from the population norms (<3% or >97%, SD 1.88) for height and weight were excluded, as were those with a BMI >27 kg/m^2 ^or <18 kg/m^2^. Pelvic markers and trunk markers were attached to volunteers, as follows. Three pelvic markers were placed on the right ASIS (anterior superior iliac spine), left ASIS, and sacrum in the middle of left and right PSIS (posterior superior iliac spine), respectively, and four trunk markers were located on the spinous process of the 7^th ^cervical vertebra, the spinous process of the 10^th ^thoracic vertebra, the jugular notch where clavicles meet the sternum, and at the xiphoid process of the sternum, respectively. Additional foot and ankle markers were placed to acquire data on gait cycles and walking speeds. Marker placement was performed as described for the Plug In Gait model (Vicon Motion Systems) [10], and was done by a single experienced operator. All subjects walked with bare feet along a 9 meter long straight lane at a self-selected speed with markers attached. Seven VICON CCD cameras (Oxford Metrics, Oxford, England) captured marker movements at a sampling rate of 60 Hz, and three trials were averaged to a single data set. For each trial (9-meter walk) one gait cycle, which was not in the initial step or in last step, was selected by one author. Three gait cycles selected from three trials were averaged to a gait cycle for one person, and the kinematic gait data was retrieved from the averaged gait cycle. The gait information obtained was processed using VICON Workstation (Version 3.1, Oxford Metrics, Oxford, England) in which Euler angle [11] was employed for the kinematic data. To display gait data, one gait cycle was represented using a 100% scale and the angular values of motions were collected at 2% intervals. The gait cycle was defined as an interval from one heel contact to the next contact made by the same heel; heel strike and toe off information was also recorded. Kinematic trunk motion data were presented for the sagittal, coronal and transverse planes, which were defined as tilt, obliquity, and rotation, respectively. For all subjects, both trunk motion in the global reference frame (motion (G), i.e., to the ground) and trunk motion in the pelvic reference frame (motion (P), i.e., relative to the plane defined by the three pelvic markers) were obtained [7,12]. We referred to motions in the three planes in those two reference frames as tilt (G), obliquity (G), rotation (G), tilt (P), obliquity (P) and rotation (P). Positive angular values were defined for forward bending in tilt, bending to the ipsilateral side in obliquity, external rotation in rotation; negative values represent the opposite movements, where the angular definition of movement in the global reference frame was converted to the opposite direction of the Euler angle [11] for a better understanding. The kinematic and basic gait data such as walking speed, cadence, and stride length were obtained separately for the right and left sides, and overall 40 sets of data were included for statistical analysis. Basic gait data were normalized by *ad hoc *normalization [13], where the data were divided by leg length or square root of leg length. Variables, such as, mean and range of trunk motion were recorded in all planes. To describe relative phase movements, we determined points of percentage in the gait cycle [14] when movement angular values were at a maximum or minimum.

### Sample size estimation and Statistical analysis

Prior sample size estimation was performed. When we assumed 5 degrees of difference between genders was significant and set standard deviations to be 2.5 degrees, sample size was calculated to be 8 subjects in each gender group (α-error 0.05, β-error 0.8).

Descriptive analysis was performed separately for all sets of data in all motion planes. Kinematic trunk motion data in global and pelvic reference frames were compared using the paired t-test or Wilcoxon's signed rank test depending on data set normality which was determined using Kolmogorov-Smirnov test. Analysis of covariance (ANCOVA) was performed to compare the kinematic variables between genders. Correlations between the trunk motion variables were evaluated using Pearson's or Spearman's correlation tests. Statistical significance was accepted for *P *values of < 0.05 except for the correlation test which was adjusted for family wise error. All statistical analyses were carried out using SPSS 11.0 (SPSS, Chicago, Illinois, USA).

## Results

Twenty volunteers were recruited for this study. Of the volunteers, 11 were male and nine were female. BMIs ranged from 18.4 kg/m^2 ^to 26.5 kg/m^2^. The heights, weights and BMIs of all subjects were between the 3 and 97 percentiles. Heights, weights, and BMIs were different between genders although ages were not significantly different. Walking speeds were not significantly different between genders, while normalized walking speeds showed significant difference between genders (p = 0.025) (Table 1).

**Table 1:** Anthropometric Data and Walking Speeds

	Male (N = 11)	Female (N = 9)	Difference	*P *value
Age (years)	31.9 (6.4)	28.6 (5.5)	3.3	0.230
Height (cm)	169.5 (3.9)	160.8 (4.4)	8.7	<0.001
Weight (kg)	68.9 (5.7)	54.4 (6.1)	14.5	<0.001
BMI (kg/m^2^)	24.0 (1.4)	21.1 (2.8)	2.9	<0.001
Walking speed (m/sec)	1.18 (0.06)	1.21 (0.09)	0.03	0.240
Walking speed/√*L*_0_	1.27 (0.07)	1.34 (0.12)	0.07	0.025
Cadence (No./min)	107.6 (4.3)	114.5 (7.6)	6.9	0.002
Cadence × √*L*_0_	100.2 (3.0)	103.8 (6.5)	3.6	0.039
Stride length (m)	1.31 (0.06)	1.26 (0.06)	0.05	0.018
Stride length/*L*_0_	1.51 (0.08)	1.54 (0.10)	0.03	0.369

*L*_0_: Leg length

### Normal values of trunk motion, comparison between trunk motion in pelvic reference frame versus global reference frame

**Figure 1:**
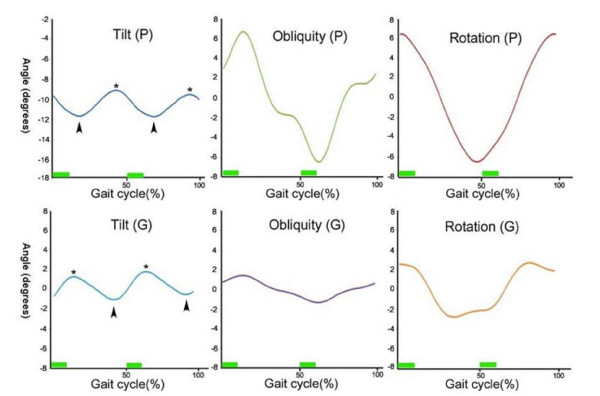
**Trunk motions in three planes**. In each graph, the transverse axis represents the phase of the gait cycle as percentages of gait cycle and the vertical axis represents angular values. The graphs depict trunk motion in each plane using global and pelvic reference frames. Relative phase of motions between two reference frames were almost reciprocal in the sagittal plane, synchronous in the coronal plane, and 15% different phase in the transverse plane. Note two repetitive motions in tilt (G) and tilt (P), and the slight differences between maxima (asterisks) and minima (arrow heads) during first and second motions, which are believed to be influenced by motions of other planes. The bars on the transverse axis represent double limb support phases.

Mean tilt (P) was about 10 degrees less than mean tilt (G), suggesting that the pelvis was anteriorly tilted at 10 degrees in the sagittal plane during gait. Mean obliquity and rotation were near 0 degrees according to both pelvic and global reference frames whilst walking, as was expected. Ranges of motions in global reference frame were smaller than those in pelvic reference frame. Range of rotation (P) was greatest and range of tilt (P) was smallest for motions in the pelvic reference frame. In terms of motions in the global reference frame, range of rotation (G) was the largest and range of obliquity (G) was the smallest (Table 2). In terms of relative phasic motion in gait cycle curves, tilt (P) and tilt (G) showed near reciprocal movement, obliquity (P) and obliquity (G) were synchronous, and rotation (P) followed rotation (G), which was delayed by 15% of the gait cycle (Figure 1).

**Table 2:** Comparison of Trunk motion (P) vs Trunk motion (G) in degrees

Motion	Value	Trunk motion (P)	Trunk motion (G)	Difference	*P *value
Trunk	Mean	-10.2 (5.5)	-0.2 (3.6)	10.0	<0.001
Tilt	Range	4.7 (2.2)	4.0 (1.8)	0.7	0.004*

Trunk	Mean	-0.0 (2.3)	-0.0 (1.3)	0.0	0.985
Obliquity	Range	13.0 (4.5)	3.3 (1.4)	9.7	<0.001

Trunk	Mean	0.1 (2.3)	-0.1 (2.1)	0.2	0.738
Rotation	Range	13.7 (4.9)	6.9 (2.9)	6.8	<0.001

*, nonparametric method (Wilcoxon signed rank test)

### Comparisons between men and women

The most prominent result was observed in the sagittal plane. Both mean tilt (P) and mean tilt (G) of women were about 5 degrees less than those of men, meaning a more extended trunk posture in women (*P *= 0.002). Ranges of tilt (P) and tilt (G) were not significantly different between gender. Range of obliquity (P) in women was larger than in men (*P *= 0.026), but no significant difference in obliquity (G) was observed between men and women, which concurred with the result of a previous study which suggested larger coronal motion of the female pelvis than male [15]. These results are detailed in Table 3. No difference in relative phase motion was observed between men and women. For the significantly different variables between genders (Table 3), an ANCOVA test was performed to exclude the confounding effect of the different BMI and normalized walking speed between genders (Table 1). The fixed factor was gender, and the covariates were the BMI and normalized speed. The dependent variables were tilt (P), tilt (G), and the range of obliquity (P). The kinematic data was found to have an equality of error variances on the Levene's test. Tilt (G) was significantly different between genders (p < 0.001) after excluding the effects of the normalized walking speed (p = 0.132) and BMI (p = 0.147) on the ANCOVA test. Tilt (P) was similar in both genders (p = 0.415), while the normalized walking speed (p = 0.004) and BMI (p = 0.040) had a significant effect on tilt (P). The range of obliquity (P) was found to be affected significantly by the normalized walking speed (p = 0.004), gender (p = 0.026), and BMI (p = 0.039).

**Table 3:** Comparison between Male (N = 11, 22 sides) and Female (N = 9, 18 sides) trunk motions (in degrees)

Motion	Value	Male	Female	Difference	*P *value
Trunk	Mean	-7.8 (5.0)	-13.0 (4.9)	5.2	0.002
Tilt (P)	Range	4.4 (2.4)	5.0 (1.7)	0.6	0.373

Trunk	Mean	-0.1 (2.6)	-0.0 (2.1)	0.0	0.954
Obliquity (P)	Range	11.6 (4.0)	14.8 (4.6)	3.1	0.026

Trunk	Mean	0.2 (2.2)	0.0 (2.4)	0.2	0.821
Rotation (P)	Range	13.9 (5.2)	14.1 (4.7)	0.2	0.598

Trunk	Mean	2.3 (2.4)	-3.1 (2.2)	5.4	<0.001
Tilt (G)	Range	4.0 (2.4)	3.9 (0.7)	0.1	0.922*

Trunk	Mean	-0.0 (1.2)	0.0 (1.5)	0.0	0.909
Obliquity (G)	Range	3.5 (1.4)	3.1 (1.3)	0.3	0.431

Trunk	Mean	-0.1 (2.1)	-0.0 (2.2)	0.1	0.914
Rotation (G)	Range	6.3 (1.7)	7.6 (3.8)	1.3	0.202

*, nonparametric method (Mann-Whitney test)

### Correlation between motion planes in trunk motion

Trunk motion (G) tended to be correlated with its counterpart trunk motion (P). Range of rotation (P) and range of obliquity (P) were found to be correlated (r = 0.617; *P *< 0.001), as were range of rotation (P) and range of obliquity (G) (r = 0.610; *P *< 0.001) (Table 4). Therefore range of trunk motion in coronal plane was correlated with that in transverse plane. In the correlation test, the number of pairs by which the alpha-error was devided was 15. Therefore, the statistical significance was set to *P *< 0.003, which was adjusted for family wise error.

**Table 4:** Correlation coefficients between Ranges of Trunk Motions

	Tilt (P)	Obliquity (P)	Rotation (P)	Tilt (G)	Obliquity (G)	Rotation (G)
Tilt (P)						
Obliquity (P)	0.054					
Rotation (P)	0.065	0.617*				
Tilt (G)	0.748*	-0.322	-0.226			
Obliquity (G)	0.412	0.495*	0.610*	0.131		
Rotation (G)	0.066	0.008	0.346	0.105	-0.033	

*, *P *< 0.003 (*P*-value adjusted for family wise error)

## Discussion

Trunk motions to the ground showed narrow ranges in all three planes, whereas trunk motions relative to the pelvis tended to be larger than those to the ground, which concurs with the results of previous studies [14,16]. Women's trunks showed 5 degrees more extended posture during gait than men's trunks. Range of trunk motion in coronal plane appeared to be correlated with range of trunk motion in transverse plane.

This study has some limitations that require consideration. First the number of cases was quite small and the generalization of our results requires confirmation by further study although prior sample size was calculated in this study. Second, our trunk model did not take the intra-truncal movement into account, and considered the trunk to be a rigid segment. The lumbar lordosis was not actually measured but calculated. The motion or posture that we considered lumbar lordosis might have originated in part from the intratruncal movement. Third, there were significant variations in the subjects' height and weight, which could affect the basic gait data. Fourth, the normalized walking speed was different between genders, which could be a confounding factor when comparing the gender differences even though we performed a ANCOVA test to excluded the different effects of BMI and normalized walking speed between genders. Fifth, the small differences between groups were statistically significant. However, these results might have been caused by variabilities of marker placement at least in part, and care should be taken when interpreting the clinical implications.

Posterior tilting of the trunk (Tilt (G) graph in Figure 1) begins with the initiation of the single limb support phase (gait cyle 10%, 60%), which is approximately the opposite movement of lower extremity during swing phase. It appears that sagittal trunk motion counterbalances the lower extremity during the single limb support phase. On the other hand, the trunk started to bend anteriorly from just before heel strike through the double limb support phase, which appears to enhance forward progression when the body is stabilized by double support. Trunk motion in the sagittal plane is two repetitive movement and each shape of the two motions in one gait cycle seems quite similar (Tilt (P) and Tilt (G) graph in Figure 1), which was also shown in other studies [4,16]. However, despite the similar shapes of the two repetitive motions, their angular values are slightly different (asterisks and arrow heads in Tilt (P) and Tilt (G) of Figure 1), because different rotation or obliquity positions caused different positions in sagittal plane. Indeed, the same degree of sagittal tilt would appear smaller than the real value in some degrees of axial rotation, and appear larger in some degrees of coronal obliquity if the rotation and obliquity were between 0 and 90 degrees. This has some implications when kinematic trunk motion data is measured or analyzed, because if the phases of gait cycles or motions in other planes are not considered at the same time, kinematic data could be distorted.

**Figure 2:**
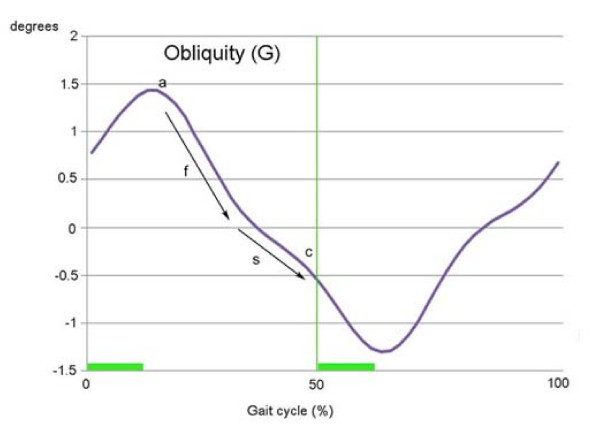
**Trunk motion in the coronal plane**. After beginning the single limb support phase (a), the trunk moves to the contralateral side (f and s). This motion decelerates slightly (s) while approaching the heel strike of the opposite foot (c, gait cycle 50%), which appears to be an effort to reduce the impact of the heel strike. The difference between the slopes of f and s represents the difference in angular velocity.

At the curve of obliquity (G) (Figure 2), the trunk starts to bend contralaterally right after the single limb support phase commenced (gait cycle 13%). During this coronal motion bending to the contralateral side, there is slightly lowered angular velocity portion (Figure 2) just before heel strike of the opposite foot (gait cycle 50%), which appears to be an effort to reduce the impact from the heel strike.

Rotational motion in the transverse plane showed the largest motion range in both the pelvic and global reference frames. A relative phase difference of 15% was observed between rotation (P) and rotation (G), which might be a means of conserving angular momentum, as was described in a previous study [17]. According to other studies [18,19], the rotational motion of trunk played an important role in adapting to the changes in walking speed. However, in this study, there was no tendency or changes in rotational motion according to the walking speed, which might be due to the relatively narrower range of walking speed than those of other studies.

On comparing the genders, no significant difference in self-selected walking speed was observed. However, after normalization, each gender showed a significantly different walking speed and cadence (Table 1). Therefore, ANCOVA test was performed to exclude the confounding effect of the different normalized walking speed and BMI between genders. The mean tilt (G) appeared to be influenced significantly by gender after eliminating the confounding effect of the normalized walking speed and BMI, while mean tilt (P) was significantly affected by normalized walking speed and BMI. The range of obliquity (P) appeared to be influenced significantly by gender, normalized walking speed and BMI. Therefore, after excluding the confounding effect of the normalized walking speed and body size, the most prominent gender difference in the kinematic data of trunk motion is believed to be the more extended trunk posture in women, which is represented by the mean tilt (G). During normal gait, women's trunks were approximately 5 degrees more extended posture than men's. A previous study [15] suggested that the female pelvis is more anteriorly tilted throughout the gait cycle, but our data showed no significant difference in mean pelvic tilt between men (mean 10.10°, SD 3.47°) and women (mean 9.89°, SD 3.82°). Therefore we believe that the 5 degrees of difference in trunk tilt between men and women came from the different lumbar lordosis, which means that women have 5 degrees more lumbar lordosis than men. This might explain the different prevalence of lumbar diseases [8,9] between genders in part through further investigation, but this topic is beyond the scope of this study.

The range of rotation (P) showed some relationship with the obliquity (P) and obliquity (G) (correlation coefficient, 0.617 and 0.610, respectively) (Table 4). We consider that sagittal trunk motion was more independent than the other two plane motions, and coronal motion and transverse plane motion are possibly interconnected in three dimensional space. This concurs with the findings of a previous study, in which coupling between lateral bending and axial rotation of the lumbar spine was suggested [20]. The vector of the spinal muscles or axis of lumbar spinal joint might explain the correlation between the coronal trunk motion and transverse trunk motion, but more study will be needed to better understand this result.

In the present study, we mainly focused on kinematic trunk motion data. More comprehensive studies, which include other body parts, kinetic data, EMG, and variations in walking speed, are recommended before we are able to understand trunk motion better. Additionally, it should be noted that some of the results of the present study differ from those of previous studies because of different numbers of cases, walking conditions (treadmill *vs*. ground walking) [21], trunk marker protocols, equipment, definitions of positive angular values of motion.

## Conclusions

Women showed 5 degrees more extended trunk posture during gait than men, which appeared to be caused by different lumber lordosis. This different lumbar lordosis could possibly explain the different prevalences of lumbar diseases between gender, which needs further investigation. Coronal trunk motion and transverse trunk motion were correlated. Kinematic trunk motion suggested its role to counterbalance the lower extremity during swing phase in sagittal plane and to reduce the angular velocity toward the contralateral side immediate before the contralateral heel strike in the coronal plane.

## Competing interests

No benefits in any form have been received or will be received from a commercial party related directly or indirectly to the subject of this article.

## Authors' contributions

All authors were fully involved in the study and preparation of the manuscript. Each of the authors has read and concurs with the content in the final manuscript. Nobody who qualifies for authorship has been omitted from the list.
